# Supplementary material for: Conditional chemoconnectomics (cCCTomics) as a strategy for efficient and conditional targeting of chemical transmission
Source: eLife. 2024 Apr 30;12:RP91927. doi: 10.7554/eLife.91927 (PMC11060718; doi:10.7554/eLife.91927)
Supplement: Figure 3—source data 3. [file elife-91927-fig3-data3.zip › Figure 3 source data3 part 2.zip!/Figure 3-Source data 3-part2/1062-13-CRISPResso_Report_jt28fhr9.zip!/CRISPResso_on_1062_16/4b.gcaggaaatgagctacctac.Insertion_deletion_substitution_locations.pdf]

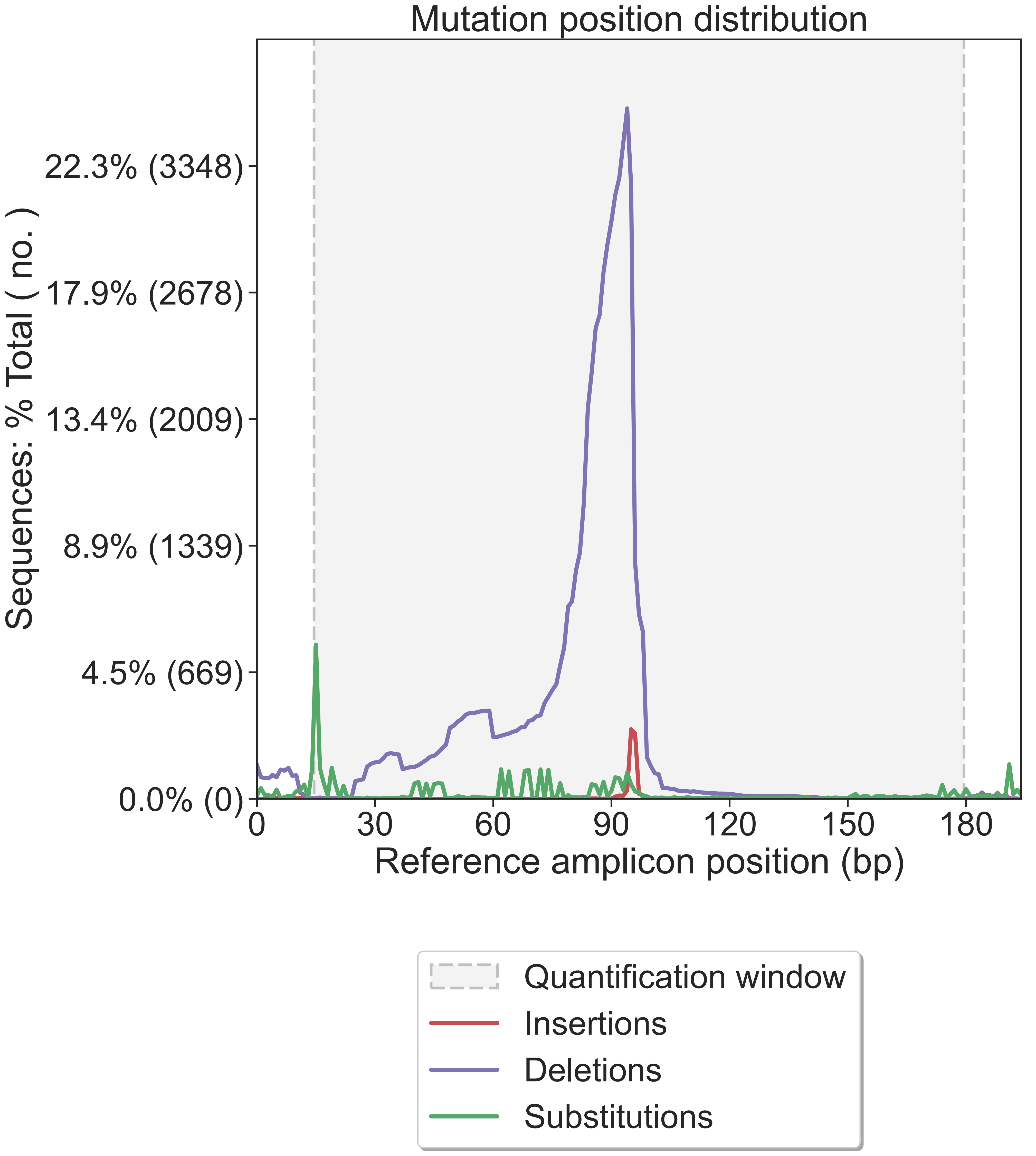

Mutation position distribution
Sequences: % Total (no.)
22.3% (3348)
17.9% (2678)
13.4% (2009)
8.9% (1339)
4.5% (669)
0.0% (0)
0
30
60
90
120
150
180
Reference amplicon position (bp)
Quantification window
Insertions
Deletions
Substitutions
